# Building Care Partner Capacity Through Education on Behaviour Change in Dementia

**DOI:** 10.1002/alz.088228

**Published:** 2025-01-09

**Authors:** Kathy Hickman

**Affiliations:** ^1^ Alzheimer Society of Ontario, Toronto, ON Canada

## Abstract

Care Partners (family and friends) providing direct care to people living with dementia are 1.6 times more likely to report distress if the person they are supporting is experiencing behaviour changes according to the Canadian Institute for Health Information. These carers are a critical part of the care team and yet they are often not provided education needed to support high quality care and more effectively contribute to the care team.

The Alzheimer Society of Ontario has developed a 6‐hour workshop (U‐First!® for Care Partners) for carers who are providing direct support to someone experiencing behaviour changes as a result of dementia or other cognitive impairment. This education is adapted from a similar program for direct health care providers and is intended to increase care partner confidence and skills to understand and respond to behaviour changes in order to i) reduce frequency and severity of behaviour changes; ii) enhance well‐being of care partners and people with dementia; iii) improve collaboration among all team members by creating common knowledge, language and approach to care. The program uses an interactive approach grounded in adult learning theory that has participants reflect on their experiences, engage with new content, practice using a variety of methods and plan how to implement what they have learned. Learners receive a participant workbook and an interactive application tool (U‐First!® Wheel), that helps them to think through potential causes of behaviour changes and supportive care strategies.

Evaluation results show lasting impacts. Even at 6 months following completion of the program, participants reported increased ability and confidence to identify, flag and respond to behaviour changes, increased ability to identify risks, increased wellbeing for themselves (including reduced stress) and for the person they are supporting. In addition, they felt the program increased their ability to have positive interactions with the person they are supporting, other care partners and health care providers. Participants with perceived high levels of stress and/or low well‐being prior to the program experienced the greatest benefits in these areas and care partner perceptions of degree of concern about behaviour changes was positively impacted by participation in the program.

